# The Subepithelial Bandlike Distribution Pattern of the CD4 Biomarker May Determine Oral Lichen Planus in the Absence of Typical Microscopic Features

**DOI:** 10.3390/ijms27114781

**Published:** 2026-05-26

**Authors:** Yang Gu, Ashley Kervin, Patricia Colp

**Affiliations:** 1 Department of Oral and Maxillofacial Sciences, Faculty of Dentistry, Dalhousie University, 5981 University Avenue, Halifax, NS B3H 4R2, Canada; a.kervin@dal.ca; 2 Department of Pathology, Faculty of Medicine, Dalhousie University, 5849 University Avenue, Halifax, NS B3H 4R2, Canada; p.colp@dal.ca

**Keywords:** oral lichen planus, oral lichenoid lesion, oral lichenoid disease, IHC, CD4, CD8, CGRP

## Abstract

Given higher compatible rates between oral lichen planus (OLP) and oral lichenoid lesions (OLLs) in histopathological and clinical features, this study aims to delineate a boundary between equivocal OLP and OLLs by biomarkers. The updated OLP diagnostic criteria in 2016 was our guideline in defining study cases of typical OLP and typical OLL with triggers, which include topical offending agents (OLL-agent), dental restorations (OLL-dental), and systemic offending drugs (OLL-drug). The expression intensity and distribution patterns of CD4, CD8, and CGRP in four groups were detected by immunohistochemistry assay (IHC). A total of 79 cases including OLP (24), OLL-agent (15), OLL-dental (21), and OLL-drug (19) were collected from an oral biopsy laboratory. Band-like distribution patterns of CD4 (100%, score 3), CD8 (54.17%, score 2), and CGRP (87.5%, score 3) in the subepithelial regions of the OLP group significantly differ from the OLL groups (each comparison pair, *p* = 0.0001). The sensitivity of CD4 (100%), specificity of CD4 (83.64%), negative predictive value of CD4 (100%), and accuracy of CD4 (83.80%) in the OLP group provide results for the diagnostic test evaluation. The band-like distribution pattern of CD4 in the subepithelial region may determine OLP when the biopsy specimen does not show typical microscopic features.

## 1. Introduction

It is challenging for oral pathologists to sign a definitive diagnosis of oral lichen planus (OLP) when the biopsy specimen does not show a typical microscopic feature that is defined as a band-like lymphocytic infiltration that demolishes the basal cell layer of hyperkeratinized stratified squamous epithelium. In 2016, the updated diagnostic criteria for OLP were posted, which were developed from the WHO 2003 modified criteria [1]. According to the new definition, a definitive diagnosis of OLP can be made only if the clinical and histopathological features both completely fit the required typical appearances. The term “histopathologically compatible with OLP” is suggested for cases where microscopic features do not make a typical appearance [1]. However, the “compatible OLP” could be an undetermined OLP or one type of oral lichenoid lesions (OLLs), which are mimicries of OLP clinically and histopathologically. In clinical practice, the OLLs are listed as differential diagnoses of OLP. Therefore, the term oral lichenoid disease was first coined in 2008 for grouping OLP and OLLs under one umbrella for the convenience of terming a similar clinical finding [2].

**Figure 1:**
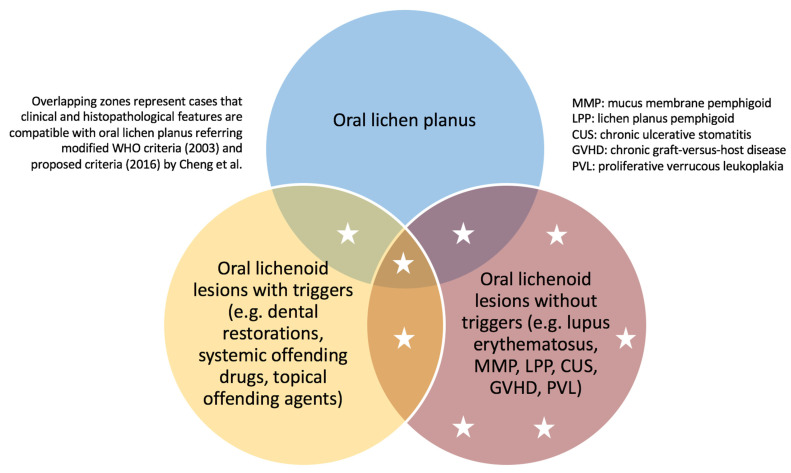
Oral lichen planus and mimicries. Non-overlapping zones represent cases of oral lichen planus (OLP) (in blue), oral lichenoid lesion (OLL) with triggers (in yellow), and OLL without triggers (in red) that have typical clinical and histopathological features. Overlapping zones include cases of OLP, OLL with triggers, and OLL without triggers that do not have typical clinical and histopathological features. The white stars outlined conditions that were excluded in this study.

Researchers investigated 85 patients diagnosed with oral lichenoid disease and found about 79% of cases were clinically typical, while about 21% were compatible; in addition, histologically, 53% of cases were classified as typical, but 47.1% as compatible [3]. This result implies that a considerable amount of undetermined OLP and OLL cases is in a “gray zone” clinically and histopathologically. Figure 1 outlines the overlaying zones of OLP and OLLs according to the list of differential diagnoses of OLP, which were reported in two position papers [1,4]. For convenience, we categorize OLLs into two groups: OLLs with triggers and OLLs without triggers. The OLLs with triggers include conditions of oral lichenoid drug reaction associated with systemic offending drugs (OLL-drug), oral lichenoid contact reaction related to dental restorations (OLL-dental), and oral lichenoid contact reaction related to topical offending agents (OLL-agent). The overlapping zones represent the “compatible OLP” and/or the “compatible OLLs”. It is crucial to delineate a boundary between equivocal OLP and OLLs after knowing the remarkable difference in malignant transformation rates (1.09% OLP and 3.2% OLL) [5] and inciting factors (absence for OLP and presence for OLL) [6]. Distinguishing two independent entities means setting different observation times for patients and informing them about the different risk of cancerization besides conducting different treatment plans. We believe there must be a method to differentiate equivocal OLP from mimicries for better treatment.

T-cell-mediated immune pathogenesis of oral lichen planus (OLP) was recognized in 1990 [7] and was further clarified in 2016 [8]. The common T-cell biomarkers are CD4, CD8, and CD3. The pioneering work had tried to identify the difference in biomarkers between OLP and OLLs before us. Table 1 summarizes the key points of biomarkers in three original research articles that were published in 1984 and 2000 [9,10,11]. No significant difference was defined between OLP and mimicries in immunohistochemistry. However, those studies were completed before the widely accepted WHO 2003 criteria. The weaknesses in the inclusive and exclusive criteria of the study cases would significantly impact their results. This gave us a chance to investigate those biomarkers again by using the updated 2016 diagnostic criteria. As an exploratory study, we do not want to be off target. We chose CD4 and CD8 biomarkers because they were related to the histopathological demonstration, while the CGRP biomarker linked with the clinical presentation [12].

The purpose of this study is to find biomarkers to differentiate OLP from mimicries. Our study methods that differ from the previous studies are: 1. using the updated 2016 diagnostic criteria of OLP as the inclusion and exclusion standards; 2. observing the expression intensity and the distribution patterns of CD4, CD8, and CGRP in situ and in simul; 3. balancing the case numbers between study groups; and 4. collecting cases of OLP and OLL with triggers that only had typical clinical and histopathological features. Excluded conditions are highlighted by white stars in Figure 1. In our view, if a biomarker works for “typical cases” (classic OLP), it should work well for “compatible cases” (equivocal OLP) as they should share the same pathogenesis.

## 2. Results

### 2.1. No Difference in the Expression Intensity Within Each Study Group

The immunohistochemistry outcome of each group was obtained by the mean value of scores in all cases of this group. Interestingly, the scores under the related distribution pattern within each group were highly consistent with a 95% confidence interval. Therefore, each group was documented by the mean score under the related distribution pattern. The immune-positive score represents the expression intensity. The expression intensity under the related distribution pattern for each biomarker is the same within each group. Herein, the score of the expression intensity was used as a grouping label, not a comparison item. Please see the first vertical column in Table 2. Herein, the comparison work in this study focuses on the difference in distribution patterns between four study groups for each biomarker.

**Figure 2:**
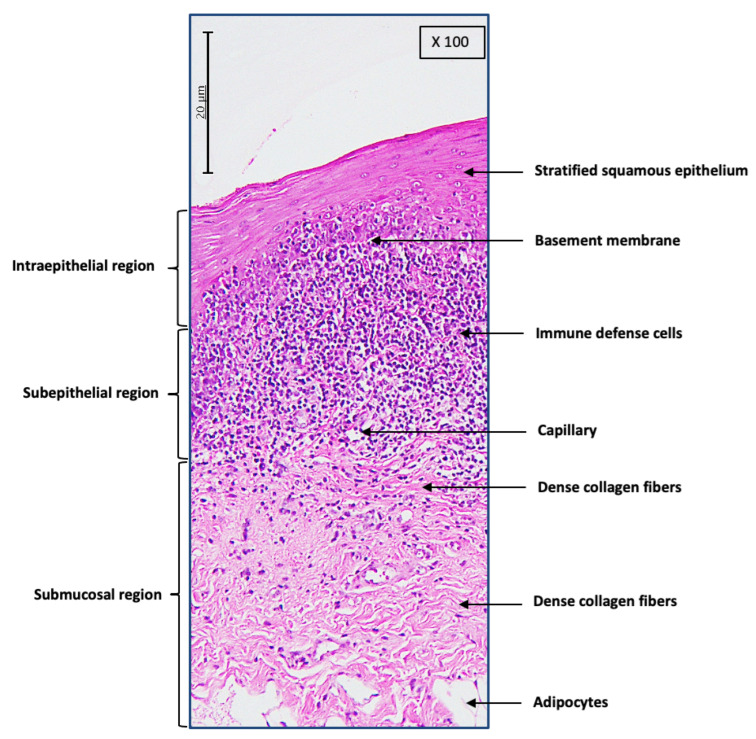
Definitions and standards of histopathological terms in the study. Definitions: “Intraepithelial region” means stratified squamous epithelial cells about the basement membrane. “Subepithelial region” means the loose connective tissue beneath the basement membrane that contains immune defense cells, lymphatic and blood capillaries, and nerve endings. “Submucosal region” means the randomly irregular dense connective tissue that contains dense collagen fibers, fibroblasts, adipocytes, minor salivary gland lobes, skeletal muscle, nerve bundles, and seldom immune defense cells depending on the anatomy site. Standards: “Intraepithelial region” is the region above the basement membrane. “Subepithelial region” is the region beneath the basement membrane and without dense collagen fibers. “Submucosal region” is the region contains dense collagen fibers.

**Figure 3:**
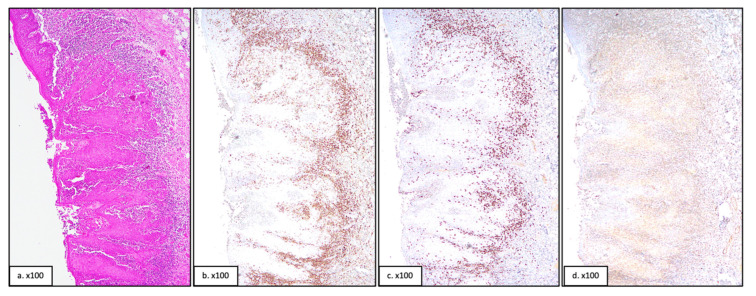
The scoring and recording baseline of the immune-positive stain for a contrast case of squamous cell carcinoma transferring from oral lichen planus. (**a**) H&E: dense subepithelial lymphohistiocytic infiltration. (**b**) CD4: score 1/3/1, band-like pattern. (**c**). CD8: score 1/2/1, patchy pattern. (**d**) CGRP: score 2/1/0, scattered pattern.

Our “Sandwich-Scoring Track” method determined the expression intensity by the immune-positive scores in the intraepithelial, subepithelial and submucosal regions for each case. Figure 2 and Figure 3 outline the scoring locations and standards. The first vertical column in Table 2 addresses three sandwich scores under three distribution patterns for three biomarkers of CD4, CD8 and CGRP.

The “Sandwich-Scoring Track” method did highlight the subepithelial region as the pivotal field to see the difference in distribution patterns between OLP and OLLs. This finding indeed provided a solid ground for the “Pattern-Based Comparison”, which was used for the following analysis.

### 2.2. Differences in Distribution Patterns of Four Study Groups

The OLP group showed a band-like distribution pattern at the subepithelial region with higher proportions in CD4 (100%, Score 3), CD8 (54.17%, Score 2), and CGRP (87.50%, Score 3). Their differences were extremely significant for three OLL groups (each comparison pair, *p* = 0.0001). In addition, the three markers were strongly correlated with the OLP group in descending order from CD4 to CGRP and CD8 based on their X^2^ values. Table 2 records the abovementioned findings.

**Figure 4:**
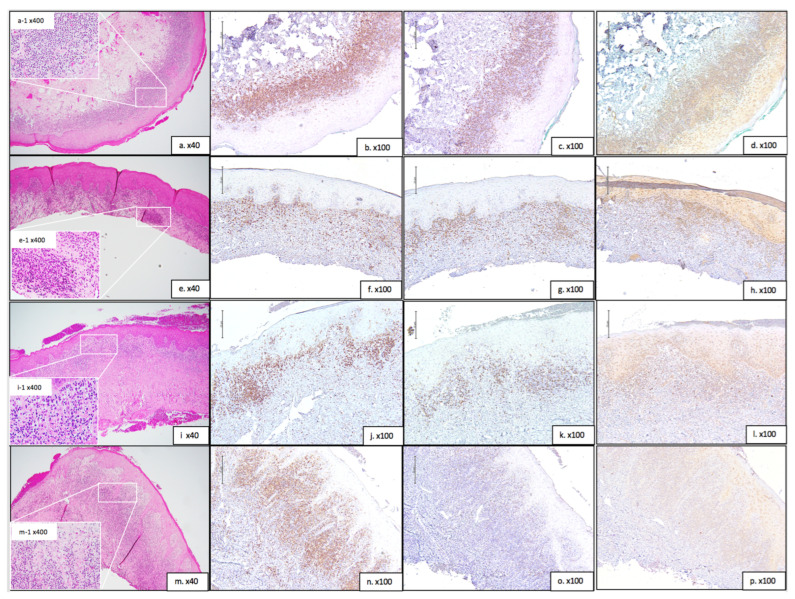
Scores and patterns of the immune-positive stain for four eligible cases. Case 1 in OLP group (**a**, a-1, **b**–**d**): (**a**) H&E ×40. a-1. H&E ×400. (**b**) CD4 ×100, score 1/3/1, band-like pattern. (**c**) CD8 ×100, score 1/2/1, band-like pattern. (**d**) CGRP ×100, score 3/3/1, band-like pattern. Case 2 in OLL-agent group (**e**, e-1, **f**–**h**): (**e**) H&E ×40. e-1. H&E ×400. (**f**) CD4 ×100, score 1/3/1, patchy pattern. (**g**) CD8 ×100, score 1/2/1, patchy pattern. (**h**) CGRP ×100, score 3/2/1, patchy pattern. Case 3 in OLL-dental group (**i**, i-1, **j**–**l**): (**i**) H&E ×40. i-1. H&E ×400. (**j**) CD4 ×100, score 1/3/1, patchy pattern. (**k**) CD8 ×100, score 1/2/1, patchy pattern. (**l**) CGRP ×100, score 3/2/1, patchy pattern. Case 4 in OLL-drug group (**m**, m-1, **n**–**p**): (**m**) H&E ×40. m-1. H&E ×400. (**n**) CD4 ×100, score 1/3/1, patchy pattern. (**o**) CD8 ×100, score 1/1/0, scattered pattern. (**p**) CGRP ×100, score 2/1/0, scattered pattern.

Surprisingly, the patchy distribution pattern of CD4 in the subepithelial region (Score 3) was prominent in all OLL groups: 93.33% in OLL-agent, 76.19% in OLL-dental, and 84.21% in OLL-drug, while 100% of the OLP cases showed the band-like distribution pattern with Score 3 (Table 2 and Figure 4).

However, subepithelial distribution patterns of CD8 and CGRP in the three OLL groups were variable. About 93.33% of OLL-agent cases with a patchy distribution pattern (Score 2) of CGRP significantly differed from both OLL-dental and OLL-drug groups (*p* = 0.0034). Further, 63.16% of OLL-drug cases with a scattered distribution pattern (Score 1) of CGRP significantly differed from both OLL-agent and OLL-dental groups (*p* = 0.0001). There was no statistically significant difference between the three OLL groups in the CD8 distribution patterns (*p* = 0.0707, *p* = 0.0677) (Table 2 and Figure 4).

### 2.3. The Diagnostic Test Evaluation

According to the prevalence (1.01%) of OLP [13] and the gold standard of the diagnostic criteria of OLP [1], a diagnostic test evaluation was performed on the data in the band-like distribution patterns of CD4, CD8, and CGRP at the subepithelial region (Table 2). The observation group (disease group) was the OLP group. The comparison group (non-disease group) covered all three OLL groups. Table 3 posts the results of sensitivity, specificity, positive predictive value, negative predictive value, and diagnostic accuracy for the three biomarkers. Their band-like distribution patterns at the subepithelial region in the OLP group were recorded by the highest rates in sensitivity (CD4 100%; CD8 54.17%), specificity (CD4 83.64%; CD8 96.36%), negative predictive value (CD4 100%; CD8 99.52%), and accuracy (CD4 83.80%; CD8, 95.94%). The other results were recorded in Table 3 as well.

## 3. Discussion

This study aims to find biomarkers to determine OLP in the absence of typical microscopic features. The methodological strategy is only collecting typical OLP cases and typical OLL cases with triggers as study samples. We believe that if a biomarker works for “typical cases”, it should work well for “equivocal cases” as they share the same pathogenesis. The typical clinical and histopathological features are clearly described in two position papers by AAOMP (2016) [1] and the WHO Collaborating Center (2021) [14]. According to the updated diagnostic criteria and inclusive standards, a total of 24 cases of OLP and 55 cases of OLL with triggers were collected. Using an immunohistochemistry assay we found the band-like distribution patterns in the subepithelial regions of CD4, CD8, and CGRP in the OLP group significantly differed from the groups of OLL with triggers (Table 2 and Figure 4). We expect the CD4 antibody could be a feasible biomarker.

### 3.1. The Band-like Distribution Pattern of CD4 in Oral Lichen Planus

CD4 is a co-receptor of T helper cells but also is found on the surface of macrophages and dendritic cells. The intensity of CD4 (Leu3a) expression within epithelium and lamina propria (another term for the subepithelial region) did not show a difference between OLP and OLL-drug cases [9]. Our study supported the conclusion since both presented with Score 3 of CD4 expression in the subepithelial regions. Further, we found the distribution pattern of CD4 in the subepithelial region was extremely different between OLP and OLL with triggers (*p* = 0.0001) (Table 2). This specific feature was additionally proved by the outstanding results in sensitivity (100%), specificity (83.64%), negative predictive value (100%), and diagnostic accuracy (83.80%) (Table 3). The CD4 antibody could help oral pathologists determine OLP when a biopsy is termed “histopathologically compatible with OLP”. This ambiguous diagnosis was frequently seen in the following circumstances: a band-like lymphohistiocytic infiltration mixed with other types of inflammatory cells (e.g., eosinophils, plasma cells), a band-like lymphohistiocytic infiltration accompanied with topical and/or systemic triggers, a continuous but not band-like lymphohistiocytic infiltration in the absence of other types of inflammatory cells, a continuous but not band-like lymphohistiocytic infiltration without any topical or systemic triggers, bullous/ulcerative forms of an oral lichenoid disease, and so on. Our findings may bring us out of the dilemma. The band-like distribution pattern of CD4 with Score 3 in the subepithelial region could be a practical standard to determine an OLP case no matter if it is a histopathologically compatible case or clinically the patient was exposed to offensive agents, dental materials, and/or drugs. We name it the “CD4 Band”.

### 3.2. The Band-like Distribution Pattern of CD8 in Oral Lichen Planus

CD8 is a co-receptor of cytotoxic T cells; additionally, it is found on the surface of natural killer cells and dendritic cells. The intensity of CD8 expression in the subepithelial region of OLP was significantly higher than the healthy control [15] and had no difference between OLP and OLL-drug cases [9]. Our study did not support the conclusion because the subepithelial intensity of CD8 was expressed in the OLP group mainly as Score 2, but in the OLL-drug group predominantly as Score 1 (Table 2). We found the band-like distribution pattern of CD8 in the subepithelial region of OLP differed from OLL with triggers (*p* = 0.0001) (Table 2), which is a specific feature for OLP due to the outstanding results in specificity (96.36%), negative predictive value (99.52%), and diagnostic accuracy (95.94%) (Table 3). However, the lower sensitivity (54.17%) renders the CD8 antibody a secondary option to reach the differentiation goal. The activated CD8 T cells greatly promote the production of Matrix Metalloproteinase 9 (MMP9) [16], which directly cleaves collagen IV resulting in the basement membrane disruption [17]. The band-like distribution pattern of CD8 in the subepithelial region of OLP may aggravate the damage to the basement membrane, but the close correlation between them shall be exploited further.

### 3.3. The Band-like Distribution Pattern of CGRP in Oral Lichen Planus

CGRP plays three roles in the inflammation region: as a nociceptive signal, as one of the most potent peripheral microvascular vasodilators, and pro- and anti-inflammatory functions depending on circumstances [12]. Besides the derivation from C and A *δ* terminal sensory fibers, skin keratinocytes produce CGRP as well [18]. Our study found the highest CGRP expression (Score 3) within the epithelium and subepithelial region only in OLP group (Table 2 and Figure 4). A pioneering work found the intensity of CGRP expression in the subepithelial region was significantly higher than the healthy control but had no difference between OLP and OLL-dental cases [11]. Unfortunately, our study did not support the conclusion because of the subepithelial intensity in the OLP group, predominantly Score 3, but mainly Score 2 in the OLL-dental group; further only the OLP group presented the band-like distribution pattern that differed from the OLL with triggers group (*p* = 0.0001) (Table 2). However, it is a challenge to account for the numbers of CGRP immune-positive cells since it is a secreted protein, not a nuclear-staining protein. As such, it is not a feasible biomarker for differentiating purposes. From another perspective, our finding seems to explain that the pain sensitivity and the erythematous base for white striations shall be more commonly noticeable in patients who have OLP compared with OLL cases. Herein, our study could be the first article to record the CGRP expression within oral epithelium. We need to point out that interpretations of CGRP and clinical symptoms such as pain sensitivity or erythematous base are interesting but should be presented as hypotheses rather than conclusions.

### 3.4. The Patchy Distribution Pattern of CD4 in Oral Lichenoid Lesions

The tertiary lymphoid follicle-like appearance in the submucosal region of oral lichenoid disease was found in 1984 [10]. This was the first immunohistochemistry assay to observe the distribution pattern of immune-positive cells for the T cell biomarker (CD3) (Table 1). Although we do not know the exact proportion of OLLs within the 82 cases of oral lichenoid diseases, the study indicated all cases close to or directly contacted with amalgam fillings, 25% (20/82) of cases with drug intake, and 3.7% (3/82) of cases with ectopic allergy. We assumed that most of the study cases were OLLs with triggers based on the current diagnostic standards. Our study found the patchy distribution pattern with the expression intensity (Score 3) of the T cell biomarker (CD4) was predominant in OLL with triggers (from 76.19% to 93.33%) (Table 2 and Figure 4). Our findings indirectly support the pioneering work referred to; the patchy distribution pattern morphologically resembled a tertiary lymphoid follicle-like appearance in the subepithelial region in the 1984 study [10].

### 3.5. The Different Distribution Patterns of CGRP Between Types of Oral Lichenoid Lesions

The patchy distribution pattern of CGRP for the OLL-agent group and the scattered distribution pattern of CGRP for the OLL-drug group are meaningful for differentiation between types of OLL with triggers. For the same reason, the difficulty of accounting for CGRP immune-positive cells, the CGRP antibody cannot be a good method to distinguish different types of OLL with triggers.

### 3.6. The Potential Contribution to the New OLP Treatment Plan

There is no doubt that OLP and OLLs are independent entities considering substantial differences in etiology, pathogenesis, and malignant transformation rate [5]. The treatment strategy of OLLs concentrates on identifying and eliminating triggers (inciting factors) [6], while empirical therapy for OLP has transferred from corticosteroids, immunosuppressants (e.g., Cyclosporin, Tacrolimus) to biologics (e.g., anti-IL-2, anti-IL-17) [19]. Our finding, the band-like distribution patterns of CD4 in the subepithelial region of OLP, probably provides scientific ground for using biologics for treating OLP. CD4 T cells after being activated and differentiated into distinct effectors (T helpers) play a major role in mediating immune response through the secretion of specific cytokines. For example, T-helper 1 cells exert the macrophage function by the secretion of IL-2, while T-helper 17 cells upgrade the neutrophil function by the secretion of IL-17. The blockage to specific cytokines directly stops the T-cell-mediated inflammation following with the tissue damage caused by macrophages and neutrophils. However, a further exploration is necessary.

### 3.7. The Limitation of the Study

We only collected 79 consecutive cases between 2018 and 2021 based on the strict inclusion and exclusion criteria of OLP and OLL with triggers. Relatively it is a small data study. Due to restricted access to data, all eligible cases came from the Oral Biopsy Service Laboratory of the Faculty of Dentistry, Dalhousie University. Moreover, this is a retrospective exploratory study. Hopefully, a large sample size study with a prospective database can repeat and reproduce the study findings.

The OLP cases in our study did not have any diagnostic systemic disease based on the available clinical records. However, the undiagnostic systemic diseases in this group cannot be excluded completely. Recent research found patients with OLP are significantly predisposed to diabetes mellitus, thyroid disease, celiac disease, liver disease, and mental conditions [20]. It is uncertain whether undiagnostic systemic diseases could impact the results of the three biomarkers in our study. Hopefully, future studies will check the related items by a laboratory examination before selecting study samples.

## 4. Materials and Methods

### 4.1. The Data Source of Samples

The Dalhousie University Research Ethics Board approved the retrospective study (No. 2020-5421). All methods were carried out in accordance with relevant guidelines and regulations. Informed consent was obtained from all subjects and/or their legal guardian(s). The study samples were retrieved from the archive in the Oral Biopsy Service Laboratory of the Faculty of Dentistry by using seven keywords that included oral lichen planus, oral lichenoid mucositis, oral lichenoid lesions, oral lichenoid reaction, oral lichenoid disease, contact stomatitis, and interface mucositis. A histotechnologist collected the biopsy specimens that were stored in the laboratory between 1 January 2018 and 31 December 2021.

**Figure 5:**
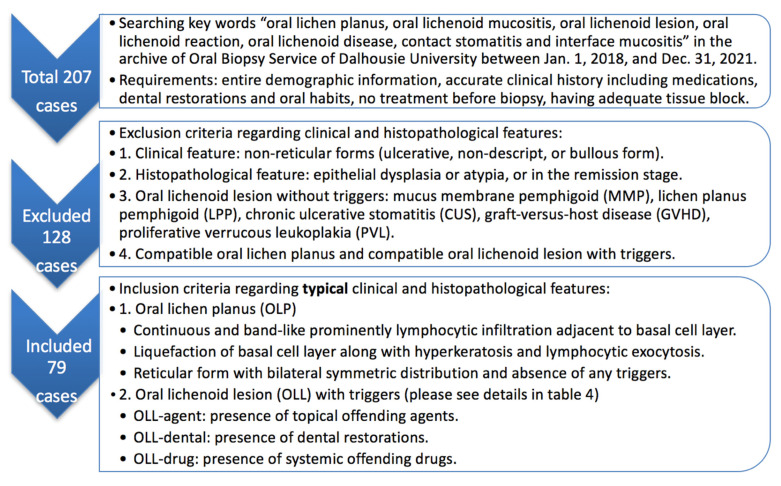
The workflow of collecting samples. The 1st step searched seven key phrases in the archive of Oral Biopsy Service. The initially included cases must meet the listed requirements. The 2nd step eliminated OLL cases without triggers, “compatible OLP” cases, and “compatible OLL with triggers” cases. Further, non-reticular forms and cases with epithelial dysplasia or atypia, or in the remission stage, were excluded. The 3rd step ensured the remaining cases that had typical clinical and histopathological features of OLP and OLL with triggers.

A total of 207 samples fulfilled the basic requirements, which were having an adequate tissue block, entire demographic information, accurate clinical history including medications, dental restorations, and oral habits of the individuals from whom the samples were taken, and no treatment performed before the biopsy. Of them, 128 samples were excluded as they met the exclusion criteria that were non-reticular forms, OLLs without triggers, “compatible OLP”, and “compatible OLL with triggers”. In addition, cases with histopathological features showing epithelial dysplasia or atypia, or in the remission stage, were excluded in the light of the following findings. A high-grade intraepithelial CD8 infiltration was seen at the remission stage of OLP [21] and the expression intensity of intraepithelial CD8 of OLP was in the same range as oral epithelial dysplasia [22]. Finally, 79 samples of typical OLPs and OLLs with triggers in the reticular form were eligible to enter the immunohistochemistry assay. Figure 5 exhibits the selection process.

### 4.2. Inclusion and Exclusion Criteria

**Figure 6:**
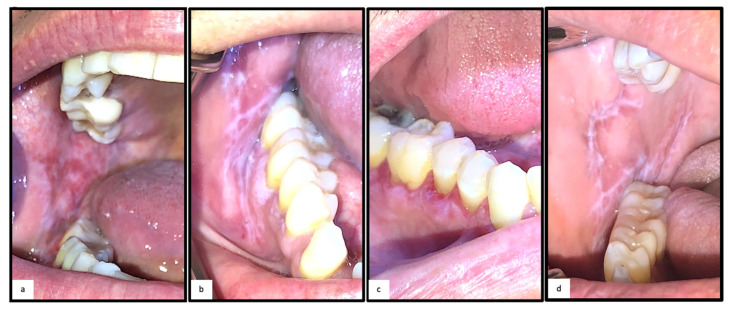
Four eligible cases presented with white reticular striations on the erythematous base. (**a**) Case 1: Oral lichen planus (OLP). (**b**) Case 2: Oral lichenoid lesion with topical offending agents (OLL-agent): exposure to cinnamon and cardamon tea for five years. (**c**) Case 3: Oral lichenoid lesion with dental restorations (OLL-dental): exposure to amalgam fillings for ten years. (**d**) Case 4: Oral lichenoid lesion with systemic offending drugs (OLL-drug): exposure to Perindopril, Candesartan, and Celebrex for seven years.

**Figure 7:**
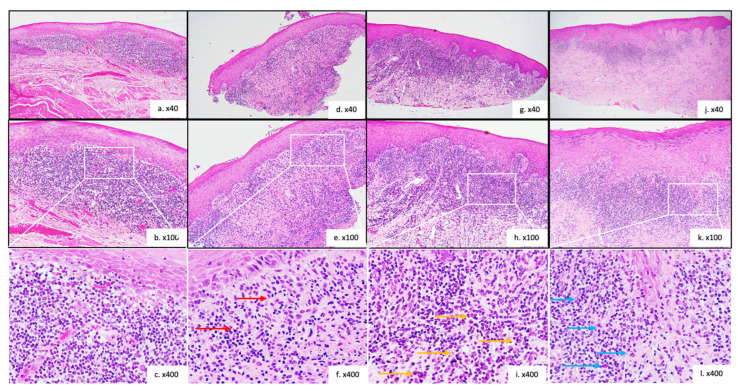
Four eligible cases with typical histopathological features (H&E). (**a**) Oral lichen planus (OLP) ×40, band-like lymphohistiocytic infiltration. (**b**) OLP ×100. (**c**) OLP ×400. (**d**) Oral lichenoid lesion-agent (OLL-agent) ×40, not band-like lymphohistiocytic infiltration with perivascular infiltration. (**e**) OLL-agent ×100. (**f**) OLL-agent ×400, arrows (red): plasma cell and eosinophil. (**g**) Oral lichenoid lesion-dental (OLL-dental) ×40, not band-like lymphohistiocytic infiltration. (**h**) OLL-dental ×100. (**i**) OLL-dental ×400, arrows (yellow): plasma cells. (**j**) Oral lichenoid lesion-drug (OLL-drug) ×40, not band-like lymphohistiocytic infiltration. (**k**) OLL-drug ×100. (**l**) OLL-drug ×400, arrows (blue): eosinophils.

Inclusion and exclusion criteria for collecting typical OLP samples were established with reference to the proposed diagnostic criteria in 2016 [1]. Since there are no widely accepted diagnostic criteria for typical OLL with triggers, we generated inclusion and exclusion criteria from the recommended standards found in the literature [1,23,24,25]. All inclusion criteria are listed one by one in Table 4 and the related images are posted in Figure 6 and Figure 7. The typical histopathological features were first described for oral lichenoid contact reaction to cinnamon (OLL-agent) in 1992 [23], for oral lichenoid contact reaction to amalgam fillings (OLL-dental) in 2006 [24], and for oral lichenoid drug reaction (OLL-drug) in 2012 [25]. Table 4 explains the details of inclusion criteria and clinical history of all eligible cases as well. Figure 6 and Figure 7 display the typical clinical features (Figure 6) and the typical histopathological features (Figure 7) of four eligible cases from each group (OLP, OLL-agent, OLL-dental, and OLL-drug) respectively. An exploratory cross-sectional study in 2021 found a mixed lichenoid inflammatory infiltrate, consisting of eosinophils and plasma cells, could be used as a reliable histopathological feature for the diagnosis of OLLs along with findings obtained from the patient’s history and clinical examination [26]. This research gave us confidence in the way of outlining the inclusion criteria for typical OLL cases with triggers.

### 4.3. Study Procedures

All eligible samples were randomly numbered in alphabetical order by the histotechnologist. The technician sectioned paraffin tissue blocks and mounted 5–8 biopsy samples on each glass slide in the Oral Biopsy Service Laboratory. All 79 study samples and two contrast cases were arranged into 12 slides named “Macro-Array Plate”. Using a manual staining procedure (Capillary Gap Technology), a chief medical laboratory technologist (PC) completed the entire immunohistochemistry procedure in the Histology and Research Service Laboratory of the Faculty of Medicine, Dalhousie University. The immunohistochemistry slides were scored and recorded by an oral pathologist and trained research assistants. To avoid scoring and recording bias, the histotechnologist who kept the original document about the diagnosis for each sample was not involved in the immune-positive scoring and recording process. The technician released the original document after the scoring and recording processes were completed and before the data analysis started.

### 4.4. Three Biomarkers in the Study

We chose CD4 and CD8 biomarkers because they were related to the pathogenesis of OLP, while the CGRP biomarker linked with the clinical presentation of OLP and OLLs. Our study samples were selected regarding the updated 2016 diagnostic criteria. CGRP as a microvascular vasodilator, neuropeptide, and immune mediator is released from sensory nerve endings when the inflammation starts [12]. The existence of CGRP assumptively may explain two typical clinical features in oral lichenoid disease: the erythematous base of white striations and the sensitivity to spicy foods. Table 1 summarizes the key points of biomarkers in three original research articles published in 1984 and 2000, even before the widely accepted WHO 2003 modified criteria. The pioneering researchers compared the difference between OLP and OLLs in the expression intensity of three biomarkers within epithelial and subepithelial regions and found no significance through an immunohistochemistry assay [9,11]. However, we would like to examine them again under the updated criteria.

### 4.5. Antibodies and Optimal Dilutions for the Immunohistochemistry Assay

The three antibodies with their immune-positive stain sites were anti-CD4 (cell membrane), anti-CD8 (secreted and cell membrane), and anti-CGRP (secreted and endoplasmic reticulum). The primary antibodies and the antibody dilution buffer were purchased from Abcam (Toronto, ON, Canada M5W 0E9), and a second antibody (mouse IgG) and reagents were bought from Inter Medico (Markham, ON, Canada). We toned the optimal antibiotic dilution in positive control specimens as recommended by Abcam. Negative controls were obtained by replacing the primary antibody with mouse IgG. The three antibodies and optimal titrated dilutions (CD4 1:500, CD8 1:400, and CGRP 1:200) are summarized and presented in Table 5.

Antigen retrieval was processed in the Biocare Autoclave by PH 6.0 citrate buffer solution (DeCloaker) immediately after deparaffinization and the enzymatic digestion was conducted by Proteinase K. The protein block and endogenous enzyme block procedures were achieved by Peroxidizer, Biocare’s background Sniper, and automation buffers (Triton-X-100 and Tween 20). The primary and secondary antibodies were washed with an automation buffer (1% BAS and 0.025% Triton X-100) after sufficient incubation times, respectively. The detection system used was a MACH 4 probe plus HRP-Polymer with DAB (diaminobenzidine) chromogen, and the counterstain was Hematoxylin.

### 4.6. Quantification of the Data

The immunohistochemistry slides were analyzed under an optical microscope (Olympus BX51 microscope; Olympus Optical Co., Tokyo, Japan) connected to a digital color camera/Q-Color 5 (Olympus). Images were obtained with 4×, 10×, and 40× objectives UPLanFI (resolution: 2.75 mm), at a size of 2560 × 1920 pixels (resolution: 1 mm = 3000 pixels) under standard conditions. Pictures were taken from the whole slide to perform image analysis.

The proportion of immune-positive cells over all cells in the high-power field (original magnification ×100) was used for observing the expression intensity. It accounts for scores 1 to 4 as follows: Score 1 ≤ 10%; Score 2 = 11–50%; Score 3 = 51–90%; and Score 4 ≥ 91%. Each case was marked by three scores in three regions separately: the intraepithelial region (above the basement membrane), the subepithelial region (beneath the basement membrane and without dense collagen fibers), and the submucosal region (containing dense collagen fibers). Please see the definitions and standards in Figure 2. We called it the “Sandwich-scoring Track”. Please see the example in Figure 3.

Further, the distribution pattern of immune-positive cells in the subepithelial region was recorded as a B pattern (band-like distribution), a P pattern (patchy distribution), and an S pattern (scattered distribution), which we called “Pattern-based Comparison”. The B pattern is defined as a constant positive staining with the even expression intensity under a medium magnification power (×100). The P pattern is defined as a continual positive staining with varying expression intensities under a medium magnification power (×100). The definition of the S pattern is circumscribed as a discontinuous positive staining with sparse expression intensities under a medium magnification power (×100). Please see the example in Figure 3.

For drawing a scoring and recording baseline, we brought two contrast cases in the immunohistochemistry assay. One was a traumatic fibroma case that did not have subepithelial inflammation and showed a negative result in the immunohistochemistry assay. The other was a squamous cell carcinoma case transferring from OLP which had a dense subepithelial lymphohistiocytic infiltration and presented with a positive result in the immunohistochemistry assay. Figure 3 illustrates the baseline of the scoring and recording method. Table 6 summarizes the scoring, recording, and demographic information for these two cases.

In total, 79 study cases in four groups successfully underwent the immunohistochemistry assay. Each case was labeled with three scores in three regions and the distribution pattern in the subepithelial region. The immunohistochemistry outcome of each group was obtained by the mean value of scores in all cases of the group. The immune-positive scores under the related distribution pattern within each group were highly consistent with a 95% confidence interval. Hence, each group was documented by the mean score under the related distribution pattern.

### 4.7. Data Analysis

The nominal data were analyzed by using Chi-square with Yates’ correction via GraphPad Prism (version 10.1.2) and setting the two-tailed significance level at *p* < 0.05. We used MedCalc software (version 22.001) to complete the diagnostic test evaluation. This index test is reported according STARD 2015 checklist for “Standards for Reporting Diagnostic Accuracy Studies”. The reference standard for evaluating the index test is the known diagnostic criteria of OLP [1]. The required prevalence (1.01%) of OLP was reported in a meta-analysis review [13].

### 4.8. The Demographic Feature Along with Some Immune-Positive Scores and Patterns

The study samples consisted of 24 cases in the OLP group, 15 cases in the OLL-agent group, 21 cases in the OLL-dental group, and 19 cases in the OLL-drug group. Table 6 unveils the demographic information along with the partial data of immune-positive scores and patterns of study samples and two contrasts. The range of age and the female ratio of study samples correspond to the demographic feature of oral lichen planus [1]. One traumatic fibroma (TF) case and one case of squamous cell carcinoma transferring from OLP (SCC-OLP) are not distributed to any study group but contribute as negative and positive contrasts. Please see Figure 3.

## 5. Conclusions

The “CD4 Band” tool may determine oral lichen planus (OLP) in the absence of typical clinical and histopathological features, which is supported by the satisfied diagnostic test evaluation. The patchy distribution pattern of CD4 in the oral lichenoid lesion (OLL) with triggers supports a pioneering work that pointed out the tertiary lymphoid follicle-like appearance in the submucosal region of OLL. The extremely strong expression of CGRP within the epithelium and subepithelial regions of OLP could possibly be interpreted as the source of pain sensitivity and the erythematous base of white striations. These findings might be meaningful to delineate a boundary between OLP and OLLs because we need to design precise treatment plans, book reasonable observation schedules, and inform different risks of cancerization with reference to their known differences in malignant transformation rates and inciting factors. This study is preliminary and interesting. The results need to be replicated in different research centers.

## Figures and Tables

**Table 1:** General comparison of previous studies in biomarkers between OLP and OLLs.

Authors	Title of the Original Research Article	Study Cases and Inclusion Criteria	Immunohistochemistry Assay	Results
Matthews et al., 1984, UK [9]	Oral lichen planus: an immuno-peroxidase study using monoclonal antibodies to lymphocyte subsets	Total 16 cases in three groups: (1) Oral lichen planus (OLP): 10. (2) Lichenoid drug reaction related to NSAIDs (OLL-drug): 3. (3) Simple keratosis: 3.	1. Monoclonal antibodies: Leu3a (CD4), Leu2a (CD8), and OKT3 (CD3). 2. Comparison: the expression intensity of staining positive cells in epithelial and subepithelial regions	1. No difference between the three groups. 2. The numbers of CD4-positive cells were predominant in all three groups.
Larsson et al., 1984, Sweden [10]	Immunohistology-chemistry of ‘tertiary lymphoid follicles’ in oral amalgam-associated lichenoid lesions	A total of 82 cases were required: (1) Diagnosis: oral lichen planus or oral lichenoid lesion (OLL). (2) Microscopic features: secondary lymphoid follicle-like structures in the submucosal region, but several cases in the lamina propria (subepithelial).	1. Monoclonal antibodies: CD3, CD79a, bcl-2, Ki67, CD68, CD21, and KiM4p. 2. Comparison: The infiltrate patterns of positive cells of study cases were compared with the pattern in secondary lymphoid follicles of tonsils.	1. The infiltrate pattern resembles the secondary lymphoid follicle. 2. All cases close to or directly contacted with amalgam fillings, 25% with drug intake, and 3. cases with atopic allergy.
Niissalo et al., 2000, UK [11]	Disorder-specific changes in innervation in oral lichen planus and lichenoid reactions	Total 19 cases in four groups: (1) Oral lichen planus (OLP): 8. (2) Amalgam-related lichenoid reaction (OLL-dental): 5. (3) Oral lichenoid reaction in remission stage: 3. (4) Healthy tissue: 3.	1. Monoclonal antibodies: Calcitonin gene-related peptide (CGRP), Substance P (SP), and other neuropeptides. 2. Comparison: the expression intensity of neuropeptides in subepithelial and submucosal regions.	1. No difference in CGRP and SP between OLP and OLL-dental groups. 2. The OLL-dental group compared with the healthy group: higher in CGRP and lower in SP.

**Note**: OLP: oral lichen planus; OLL: oral lichenoid lesion; NSAIDs: non-steroid anti-inflammation drugs; CGRP: Calcitonin gene-related peptide; SP: Substance P.

**Table 2:** The immunohistochemistry outcomes for the expression intensity and distribution patterns in four study groups.

Score ^1^; Pattern ^2^	OLP Group (24 Cases)	OLL-Agent Group (15 Cases)	OLL-Dental Group (21 Cases)	OLL-Drug Group (19 Cases)	Comparison Pair; Chi-Square with Yates’ Correlation
	Ratio (%)	Ratio (%)	Ratio (%)	Ratio (%)	
CD4					
1/3/1; Band-like distribution	24/24 (100)	1/15 (6.67)	5/21 (23.81)	3/19 (15.79)	OLP vs. all OLLs; X^2^ = 44.676, *p* = 0.0001
1/3/1; Patchy distribution	0/24 (0)	14/15 (93.33)	16/21 (76.19)	16/19 (84.21)	OLL-agent vs. other OLLs; X^2^ = 0.610, *p* = 0.4347
CD8					
1/2/1; Band-like distribution	13/24 (54.17)	1/15 (6.67)	1/21 (4.76)	0/19 (0)	OLP vs. all OLLs; X^2^ = 24.548, *p* = 0.0001
1/2/1; Patchy distribution	8/24 (33.33)	4/15 (26.66)	10/21 (47.62)	3/19 (15.79)	OLL-dental vs. other OLLs; X^2^ = 3.266, *p* = 0.0707
1/1/0; Scattered distribution	3/24 (12.50)	10/15 (66.67)	10/21 (47.62)	16/19 (84.21)	OLL-drug vs. other OLLs; X^2^ = 3.338, *p* = 0.0677
CGRP					
3/3/1; Band-like distribution	21/24 (87.50)	1/15 (6.67)	5/21 (23.81)	3/19 (15.79)	OLP vs. all OLLs; X^2^ = 32.941, *p* = 0.0001
3/2/1; Patchy distribution	2/24 (8.33)	14/15 (93.33)	14/21 (66.67)	4/19 (21.05)	OLL-agent vs. other OLLs; X^2^ = 8.582, *p* = 0.0034
2/1/0; Scattered distribution	1/24 (4.17)	0/15 (0)	2/21 (9.52)	12/19 (63.16)	OLL-drug vs. other OLLs; X^2^ = 18.817, *p* = 0.0001

**Note:** ^1^ Score: intraepithelial/subepithelial region/submucosal region. ^2^ Pattern: subepithelial distribution pattern. OLP: oral lichen planus; OLL: oral lichenoid lesion; CD4: anti-CD4 antibody; CD8: anti-CD8 antibody; CGRP: Calcitonin gene-related peptide.

**Table 3:** The results of the diagnostic test evaluation for the band-like distribution patterns of three biomarkers in the OLP group.

Biomarkers in OLP as B Pattern	Sensitivity, 95% CI	Specificity, 95% CI	Positive Predictive Value, 95% CI	Negative Predictive Value, 95% CI	Likelihood Ratio, 95% CI	Accuracy, 95% CI
CD4	100%, 85.75–100%	83.64%, 71.20–92.23%	5.87%, 3.32–10.18%	100% N/A	Positive 6.11, 3.36–11.11 Negative 0.00, N/A	83.80%, 73.80–91.13%
CGRP	87.50%, 67.64–97.32%	83.64%, 71.20–92.23%	5.17%, 2.86–9.18%	99.85%, 99.56–99.95%	Positive 5.35, 2.89–9.90 Negative 0.15, 0.05–0.43	83.68%, 73.66–91.04%
CD8	54.17%, 32.82–74.45%	96.36%, 87.47–99.56%	13.19%, 3.58–38.35%	99.52%, 99.25–99.69%	Positive 14.9, 3.64–60.98 Negative 0.48, 0.31–0.74	95.94%, 88.93–99.09%

**Note:** 95% CI: 95% confidence intervals; OLP: oral lichen planus; CD4: anti-CD4 antibody; CD8: antiCD8 antibody; CGRP: Calcitonin gene-related peptide; B pattern: band-like distribution pattern.

**Table 4:** Inclusion criteria and clinical information of four study groups.

Groups	Typical Clinical Features	Typical Histopathological Features	Clinical History Including Triggers	Cases
OLP group [1]	Bilateral, symmetric, white reticular striations on erythematous bases	1. Continuous and band-like prominently lymphocytic infiltration adjacent to the basal cell layer, which has liquefactive degeneration. 2. Epithelial hyperkeratosis and intraepithelial lymphocytic exocytosis. 3. No plasma cells, no eosinophils, and no perivascular infiltration.	Idiopathic lesions without topical and systemic triggers.	24
OLL-agent group [1,23]	1. Bilateral or unilateral, white reticular striations on erythematous bases. 2. The lesion directly contacted the offending agents for over three months.	1. Continuous, but not band-like lymphohistiocytic infiltration adjacent to basal cell layer, which has liquefactive degeneration. 2. Epithelial hyperkeratosis and intraepithelial lymphocytic exocytosis. 3. The infiltration is mixed with plasma cells and eosinophils. 4. Perivascular infiltration.	1. Non-commercial wines. 2. Some commercial mouth rinses. 3. Some commercial toothpaste. 4. Some commercial denture relining material. 5. Sunflower seeds with shells. 6. Cinnamon, Mint, and Cardamon. 7. Cannabis and Tobacco.	15
OLL-dental group [1,24]	1. Bilateral or unilateral white reticular striations on erythematous bases. 2. The lesion directly contacted the dental restorative materials for over one year.	1. Continuous, but not band-like lymphohistiocytic infiltration adjacent to basal cell layer, which has liquefactive degeneration. 2. Epithelial hyperkeratosis and intraepithelial lymphocytic exocytosis. 3. The infiltration is mixed with prominent plasma cells.	1. Amalgam fillings. 2. Porcelain-fused-to-metal crowns (PFM). 3. Gold crowns.	21
OLL-drug group [1,25]	1. Bilateral, asymmetric, white reticular striations on erythematous bases. 2. The patient has been taking the offending drugs for over one year.	1. Continuous, but not band-like lymphohistiocytic infiltration adjacent to basal cell layer, which has liquefactive degeneration. 2. Epithelial hyperkeratosis and intraepithelial lymphocytic exocytosis. 3. The infiltration is mixed with prominent eosinophils.	1. Antihypertension: Olmesartan, Candesartan, Perindopril, Trandopril, Monopril, Quinapril, Felodipine, Diltiazem, Metoprolol, Bisoprolol. 2. Non-steroid anti-inflammatory drugs: Celebrex, Ibuprofen. 3. Bisphosphonate: Alendronate. 4. Anti-lipid: Atorvastatin and Rosuvastatin. 5. Anti-allergy: Cetirizine. 6. Anti-epileptic: Lamotrigine, Clonazepam. 7. Anti-acid: Rabeprazole. 8. Antibiotics: Macrobid.	19

**Note:** OLL-agent: oral lichenoid contact reaction related to topical offending agents; OLL-dental: oral lichenoid contact reaction related to dental restorations; OLL-drug: oral lichenoid drug reaction associated with systemic offending drugs.

**Table 5:** Three antibodies and optimal titrated dilutions for the immunohistochemistry assay.

Antibody	CD4	CD8	CGRP
Abcam Number	Ab133616	Ab4055	Ab47027
Primary antibody	Rabbit monoclonal	Rabbit polyclonal	Rabbit polyclonal
Positive control	Tonsil	Tonsil	Brain cortex
Immune-positive stain site	Cell membrane	Secreted & cell membrane	Secreted & endoplasmic reticulum
Optimal dilution	1:500	1:400	1:200

**Note:** CD4: anti-CD4 antibody; CD8: anti-CD8 antibody; CGRP: Calcitonin gene-related peptide.

**Table 6:** The demographic information along with some immune-positive scores and patterns.

	Cases	Age ^1^ (Average)	Female: Male	CD4 Score ^2^; Pattern ^3^	CD8 Score; Pattern	CGRP Score; Pattern
TF	1	50	1:0	Barely	Zero	Zero
SCC-OLP	1	74	0:1	1/3/1; band-like	1/2/1; patchy	2/1/0; scattered
OLP	24	34–74 (59.9)	12:12	1/3/1; band-like	1/2/1; band-like	3/3/1; band-like
OLL-agent	15	37–77 (59.8)	7:8	N/A ^4^	N/A	N/A
OLL-dental	21	50–81 (63.8)	15:6	N/A	N/A	N/A
OLL-drug	19	30–72 (53.5)	10:9	N/A	N/A	N/A

**Note:** TF: traumatic fibroma; SCC-OLP: squamous cell carcinoma transferring from oral lichen planus; OLP: oral lichen planus; OLL: oral lichenoid lesion; CD4: anti-CD4 antibody; CD8: anti-CD8 antibody; CGRP: Calcitonin gene-related peptide. ^1^ Age: years old. ^2^ Score: intraepithelial/subepithelial region/submucosal region; please see the denoted standard in the 2.5 and 3.1 text. ^3^ Pattern: subepithelial distribution pattern; please see the denoted standard in the 2.5 and 3.1 text. ^4^ N/A: Please see details in Table 2.

## Data Availability

The data presented in this study are available on request from the corresponding author due to her current retirement status. This research and the article were completed before her retirement.
